# Intraoperative bowel perfusion assessment methods and their effects on anastomotic leak rates: meta-analysis

**DOI:** 10.1093/bjs/znad154

**Published:** 2023-05-30

**Authors:** Maxwell S Renna, Mariusz T Grzeda, James Bailey, Alison Hainsworth, Sebastien Ourselin, Michael Ebner, Tom Vercauteren, Alexis Schizas, Jonathan Shapey

**Affiliations:** School of Biomedical Engineering and Imaging Sciences, King’s College London, London, UK; Department of General Surgery, Guy’s and St Thomas’ NHS Foundation Trust, London, UK; School of Biomedical Engineering and Imaging Sciences, King’s College London, London, UK; Department of General Surgery, University of Nottingham, Nottingham, UK; Department of General Surgery, Guy’s and St Thomas’ NHS Foundation Trust, London, UK; School of Biomedical Engineering and Imaging Sciences, King’s College London, London, UK; Hypervision Surgical Ltd, London, UK; Hypervision Surgical Ltd, London, UK; School of Biomedical Engineering and Imaging Sciences, King’s College London, London, UK; Hypervision Surgical Ltd, London, UK; Department of General Surgery, Guy’s and St Thomas’ NHS Foundation Trust, London, UK; School of Biomedical Engineering and Imaging Sciences, King’s College London, London, UK; Hypervision Surgical Ltd, London, UK; Department of Neurosurgery, King’s College Hospital, London, UK

## Abstract

**Background:**

Anastomotic leak is one of the most feared complications of colorectal surgery, and probably linked to poor blood supply to the anastomotic site. Several technologies have been described for intraoperative assessment of bowel perfusion. This systematic review and meta-analysis aimed to evaluate the most frequently used bowel perfusion assessment modalities in elective colorectal procedures, and to assess their associated risk of anastomotic leak. Technologies included indocyanine green fluorescence angiography, diffuse reflectance spectroscopy, laser speckle contrast imaging, and hyperspectral imaging.

**Methods:**

The review was preregistered with PROSPERO (CRD42021297299). A comprehensive literature search was performed using Embase, MEDLINE, Cochrane Library, Scopus, and Web of Science. The final search was undertaken on 29 July 2022. Data were extracted by two reviewers and the MINORS criteria were applied to assess the risk of bias.

**Results:**

Some 66 eligible studies involving 11 560 participants were included. Indocyanine green fluorescence angiography was most used with 10 789 participants, followed by diffuse reflectance spectroscopy with 321, hyperspectral imaging with 265, and laser speckle contrast imaging with 185. In the meta-analysis, the total pooled effect of an intervention on anastomotic leak was 0.05 (95 per cent c.i. 0.04 to 0.07) in comparison with 0.10 (0.08 to 0.12) without. Use of indocyanine green fluorescence angiography, hyperspectral imaging, or laser speckle contrast imaging was associated with a significant reduction in anastomotic leak.

**Conclusion:**

Bowel perfusion assessment reduced the incidence of anastomotic leak, with intraoperative indocyanine green fluorescence angiography, hyperspectral imaging, and laser speckle contrast imaging all demonstrating comparable results.

## Teaser

Anastomotic leak (AL) is one of the most feared complications of colorectal surgery and strongly linked to poor blood supply to the anastomotic site. Several technologies have been developed for intraoperative assessment of the bowel’s blood supply. This study found that using an intervention to measure bowel perfusion reduced the rate of AL. Indocyanine green fluorescence angiography, hyperspectral imaging, and laser speckle contrast imaging demonstrated comparable results, and an ability to reduce the risk of AL when used during surgery. The field would benefit from a large-scale RCT comparing perfusion assessment modalities.

## Background

Colorectal cancer is a common cancer with an increasing incidence^1,2^. For most patients, potentially curative treatment requires major surgical resection of the affected bowel. In England and Wales in 2018, this equated to around 19 000 of the 31 000 cancers diagnosed^3^. After resection, when feasible, it is preferable to anastomose the remaining bowel^4^. It is generally accepted that a good blood supply is required to allow anastomotic healing. With inadequate blood supply, the anastomosis is likely to leak^5,6^.

Anastomotic leakage is a serious complication of colorectal resection associated with a significant increase in mortality and morbidity at 30 days after operation and beyond^7^. Leak rates in colorectal surgery are estimated at around 1–19 per cent and have a mortality rate of up to 35 per cent, depending on both patient and operative factors^7^. The relationship between hypoperfusion and an increased incidence of anastomotic leakage has been well documented, and is probably the most important risk factor for leakage^5^.

Several intraoperative techniques to measure tissue perfusion at the site of anastomosis have been described. One of the most researched methods is indocyanine green fluorescence angiography (ICG-FA). This procedure requires the injection of a dye and subjective intraoperative assessment of perfusion by the surgeon. Laser speckle contrast imaging (LSCI) is another technique described, whereby the movement of blood within the tissue changes the observed laser speckle pattern projected by the device, producing a contrast agent-free measurement of perfusion^5^. Another method gaining prominence is hyperspectral imaging (HSI). HSI does not require the use of any specific contrast product, but can offer a real-time analysis of perfusion using visible light, as well as near-infrared light in one commercial system This system enables each pixel to be analysed to provide an estimation of local tissue oxygenation^8,9^. A final perfusion method is diffuse reflectance spectroscopy (DRS), which analyses diffuse reflected light at discrete pinpoint locations in contact with the serosa of the bowel to identify colonic oxygen saturations^10^.

The aim of this systematic review and meta-analysis was to assess the various types of intraoperative perfusion measurement and evaluate their impact on anastomotic leak rates after surgery.

## Methods

### Study design

This was a systematic review of all available data on the use of intraoperative bowel perfusion imaging during colorectal surgery. The study was registered with PROSPERO before initiation of searches (CRD42021297299, 9 December 2021). Expert surgeons, librarians, and statisticians were consulted on the study design, search methodology, and statistical analysis.

### Inclusion and exclusion criteria

RCTs and comparative studies that used intraoperative perfusion assessment methods to identify bowel perfusion, and documented the rates of anastomotic leakage after surgery, were included. Procedures performed must have been elective and were not limited to minimally invasive surgery. All studies must have been clinical and related to humans, with both demographic and anastomotic leakage data available. A time limit of 21 years (2001–2022) was used to ensure that only relevant studies of intraoperative perfusion imaging were included in what is a relatively new intervention, first described in 1993^11^. Participants in the studies must have been adults with a colorectal pathology requiring intervention. Only English-language studies were included. Studies were excluded if full articles were not accessible or they were systematic reviews or case reports. Where studies used multiple methods of perfusion assessment, only the modality that was used first was included as this was most likely to influence the decision regarding anastomotic site.

### Outcomes

The primary outcome was the incidence of anastomotic leak within 30 days of major colorectal surgery involving intraoperative use of bowel perfusion imaging. Secondary outcomes included anastomotic site location changes, tissue oxygenation measurements, threshold of perfusion cut-offs, and sensitivities.

### Search strategy

Sources searched included Embase, MEDLINE, Cochrane Library, Scopus, and Web of Science. The first literature search was undertaken on 28 December 2021, with subsequent searches carried out on 1 March 2021 and 29 July 2022. Searches were supplemented by reviews of studies included in relevant systematic reviews to that ensure all available studies were included. The following Boolean search terms were applied: (Ascending Colon OR Colon OR Sigmoid OR Colorectal Surgery OR Bowel) AND (Surgical Anastomosis OR Postoperative Complications OR Anastomotic Leak OR Postoperative Complications) AND (Perfusion OR Perfusion Index OR Perfusion Imaging OR Ischemia OR Imagery).

### Data extraction

A data extraction template was completed by two researchers to collect study-level information for each study meeting the inclusion criteria. Data collected included: study data (study design, year, country), demographic data (number of patients included, age, sex, BMI), outcome data (operative focus, number of anastomotic leaks, perfusion modality used, site relocation data, definition of anastomotic leak used), and device data (specific device used, sensitivity, perfusion threshold). Any disagreements between reviewers were resolved through discussion. Where a case–control methodology had been used, the total number of participants was included as well as the case and control numbers.

### Assessment of risk of bias in included studies

Methodological quality was assessed using the Methodological Index for Non-Randomized Studies (MINORS) scoring system^12^. The MINORS criteria were chosen to enable the assessment of both randomized and non-randomized studies. The MINORS criteria were modified to fit the characteristics of included studies; scoring criteria can be seen in the *supplementary material*. Publication bias was assessed using a funnel plot (*supplementary material*).

### Statistical analysis

Results were subjected to a meta-analysis in which the main findings from the studies were combined and synthesized^13,14^. The main outcomes analysed were the number of patients with anastomotic leaks expressed as proportions of the total number of patients observed. A meta-analysis was performed using both a common-effect and random-effects models^13^. The estimation of effects and their confidence intervals was conducted on proportions transformed to logit units^15^; once estimations had been obtained, for reporting purposes, the results were converted back to the original units (proportions) to ease interpretation. Forest plots were produced to illustrate the results. Interstudy variance was estimated using the DerSimonian and Laird method^16^ as recommended by Wang^15^. Summary effect sizes were estimated as weighted means of the observed effects of individual studies. Once the results had been obtained, sensitivity analysis was performed. The magnitude of heterogeneity of study effects was quantified using the level of between-study variance represented by τ^2^. Q statistics were used, which form part of the formal test of the null hypothesis stating that τ^2^ = 0, with *P* values also reported in forest plots. *P* < 0.050 was considered to indicate a significant level of heterogeneity between studies. Heterogeneity was also measured in terms of the *I*^2^ index, which indicates the percentage of the total variability accounted for by between-study variance^13^. Significant levels of study heterogeneity were defined by values of *I*^2^ exceeding 50 per cent^17^. All the above mentioned indices and *P* values (Q statistics) were reviewed together as summary information from which the conclusions about the existence of an important level of heterogeneity among study effects were derived. All analyses were undertaken in R version 3.6.1 (R Foundation for Statistical Computing, Vienna, Austria).

## Results

### Study characteristics

**Fig. 1:**
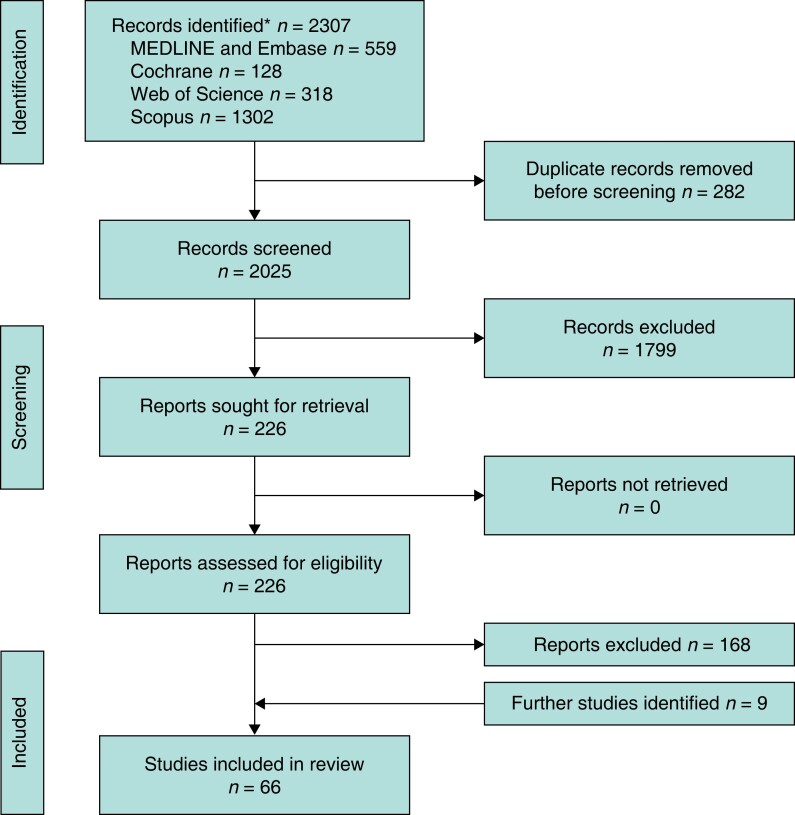
PRISMA flow diagram showing selection of articles for review †Reasons for exclusion listed in *supplementary material.*

The initial search yielded 2307 studies. After removal of 282 duplicates, the titles and abstracts of the remaining 2025 records were screened for appropriateness and a further 1799 were excluded. A total of 226 studies were retrieved for full-text review by two independent researchers and 168 studies were excluded. After repeated searching, eight more studies were added and a total of 66 studies were included in this systematic review (*Fig. 1*)^5–81,82^. Related recent systematic reviews^83–90^ on intraoperative perfusion were also reviewed to ensure that no studies had been missed.

The 66 included studies included data from 11 560 patients. A summary of each study, including patient characteristics, can be found in the *supplementary material*. Fifty-six studies were from a single centre and 10 were multicentre in design. Included studies were from a total of 17 nations and 1 study spanned multiple nations. Seventeen studies took place in Japan, 10 in the USA, 10 in Italy, 8 in Germany, and 3 in China; 2 or fewer were conducted by other nations.

### Intraoperative perfusion measuring modalities in current use

The searches identified four methods of assessing intraoperative colonic perfusion that are currently in use. The most assessed intervention was ICG-FA with 10 789 patients enrolled in 52 studies, followed by DRS with 321 patients across 6 studies, HSI with 265 patients across 5 studies, and LSCI with 185 patients across 3 studies. ICG-FA uses near-infrared technology to fluoresce ICG. DRS uses diffuse light reflectance technology in contact with the bowel at discrete locations to give a serosal tissue oxygenation (*S*to_2_) value. LSCI uses speckle patterns and flow to assess perfusion at a distance using a camera system. HSI uses reflection of a light source to assess colonic perfusion (also *S*to_2_) at a distance. ICG-FA, HSI, and LSCI rely on blood flow to generate perfusion data, whereas DRS measures oxygenated and deoxygenated blood directly.

An overview of the devices used, along with the benefits and drawbacks of each technology, taken from the manufacturer’s information where available, is shown in *Table 1*.

**Table 1:** Benefits and drawbacks of technologies included in this review

Modality	Devices used	Benefits	Drawbacks
ICG-FA	Firefly™ robotic surgical system, Intuitive Surgical (L) Photodynamic Eye© PC6100 C9830–10, Hamamatsu (O) Novadaq SPY, Stryker© (O) SPY Elite System, Stryker© (O) PINPOINT Endoscopic Fluorescence Imaging System, Stryker© (L) 1588 Advanced Imaging Modalities, Stryker© (L) D-light P system, Karl Storz© (L) IC-View, Pulsion Medical Systems© (O) VISERA ELITE2 system, Olympus© (L) Opto-cam 2100, Optomedic (L) HyperEye Medical System, Mizuho Medical Company (O) The Quest Artemis, Quest Medical Imaging© (O) VisionSense™ VS Iridium, Medtronic (L)	Visual markers of well perfused areas Many studies evaluating its use Many devices available	Lack of objective marker of blood supply Requires dark operating environment if not laparoscopic Requires injection of dye No standardized protocol, concentration, uptake time
DRS	O2C, LEA-Medizintechnik© (O) T-Stat, Spectros Corporation© (L) IntraOx device, ViOptix© (L) INVOS, Medtronic© (L)	Gives quantitative value for oxygen levels No need for medications	Can only monitor a very localized area of bowel at one time Requires tissue contact Repeat measurements required across bowel for clinical decision-making for wide-field analysis Not all included devices have been cleared for commercial use Few validated studies
HSI	TIVITA^®^ Tissue system, Diaspective Vision© (O)	No medications required Can provide objective measurement of *S*to_2_ visually and numerically	Current systems not real time Open surgery system requires background light to be turned off Few validated studies Included system not laparoscopic
LSCI	LSFG device, Softcare Co© (L) MoorFLPI-2, Moor Instruments© (L)	No medication required Demonstrates non-quantitative blood flow	System susceptible to tissue/imaging device motion Included systems not real time and images must be superimposed on an existing image Few validated studies

O, device used only for open surgery; L, device can be used laparoscopically. ICG-FA, indocyanine green fluorescence angiography; DRS, diffuse reflectance spectroscopy; HSI, hyperspectral imaging; *S*to_2_, serosal tissue oxygenation; LSCI, laser speckle contrast imaging. Further details of devices can be found in *supplementary material*.

Intuitive Surgical (Sunnyvale, California (CA), United States), Hamamatsu (Shizuoka, Japan), Stryker (Kalamazoo, Michigan (Mich), United States), Karl Storz (Tuttlingen, Germany), Pulsion Medical Systems (Midlothian, UK), Olympus (Tokyo, Japan), Optomedic (Guangdong, China), HyperEye Medical System, Mizuho Medical Co. (Tokyo, Japan), Quest Medical Imaging (Wieringerwerf, The Netherlands), LEA-Medizintechnik (Tuttlingen, Germany), Spectros Corp. (Texas, TA, USA), ViOptix Inc (Newark, CA, United States), Medtronic (Dublin, Ireland), Diaspective Vision GmbH (Salzhaff, Germany), Softcare Co. (Japan), Moor Instruments (Devon, UK).

### Outcome assessment

The primary outcome was the variation in anastomotic leak rates across the different perfusion methods. The overall pooled incidence of anastomotic leak was 7.4 per cent across the four included groups when perfusion measurements were used, compared with 12.4 per cent in the control groups (*Table 2*).

**Table 2:** Anastomotic leak rates across four perfusion measurement modalities

Modality	No. of articles	No. of participants	No. of cases	No. of controls	No. with AL*		Site relocation cases*
					Cases	Controls	
ICG-FA	52	10 789	5739	5050	238 (4.1)	454 (9)	533 (10.7)
DRS	6	321	321	0	43 (13.4)	–	3 (9.4)
HSI	5	265	265	0	21 (7.9)	–	30 (60)
LSCI	3	185	71	114	3 (4.3)	18 (15.8)	0 (0)
Total	66	11 560	6396	5164	305 (7.4)†	472 (12.4)†	566 (20)†

Values are *n* (%). †Average as opposed to total. Site relocation data consider only studies that documented the parameter. AL, anastomotic leak; ICG-FA, indocyanine green fluorescence angiography; DRS, diffuse reflectance spectroscopy; HSI, hyperspectral imaging; LSCI, laser speckle contrast imaging. Percentages may not total 100% due to rounding.

Secondary outcomes included the rates of reoperation to resite the anastomosis, tissue oxygenation measurements, and threshold of perfusion cut-offs. The rate of resiting of the anastomotic transection margin and anastomosis ranged between 0 and 100 per cent across all groups within studies. DRS and ICG-FA were associated with similar relocation rates of 9.38 and 10.69 per cent respectively for studies that included these data. The impact of intraoperative assessment of tissue perfusion on duration of operation was reported infrequently. Of 21 ICG-FA studies, the mean increase in operating time was 5.4 (range –22 to 38.1) min. Only one LSCI study^50^ reported operating times, and documented an average increase of 56 min with intraoperative use of the technology. Reoperation rates were also variable across the groups. The reoperation rate was 14 per cent across 2 DRS studies, 0 per cent across 2 LSCI studies, and 4 per cent across 28 studies in the ICG-FA group. Reoperation rates were not reported for the HSI studies.

### Meta-analysis

**Fig. 2:**
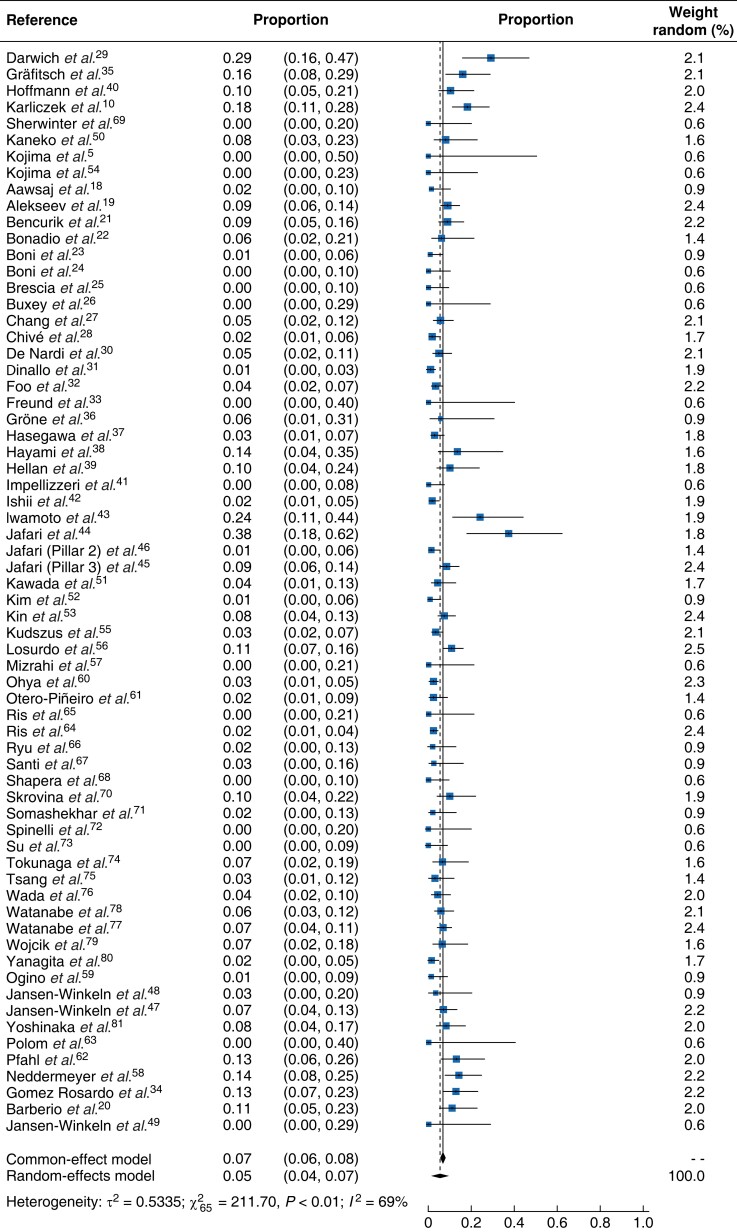
Forest plot with all studies included (cases only) demonstrating risk of anastomotic leak within 30 days Proportions are shown with 95 per cent confidence intervals.

**Fig. 3:**
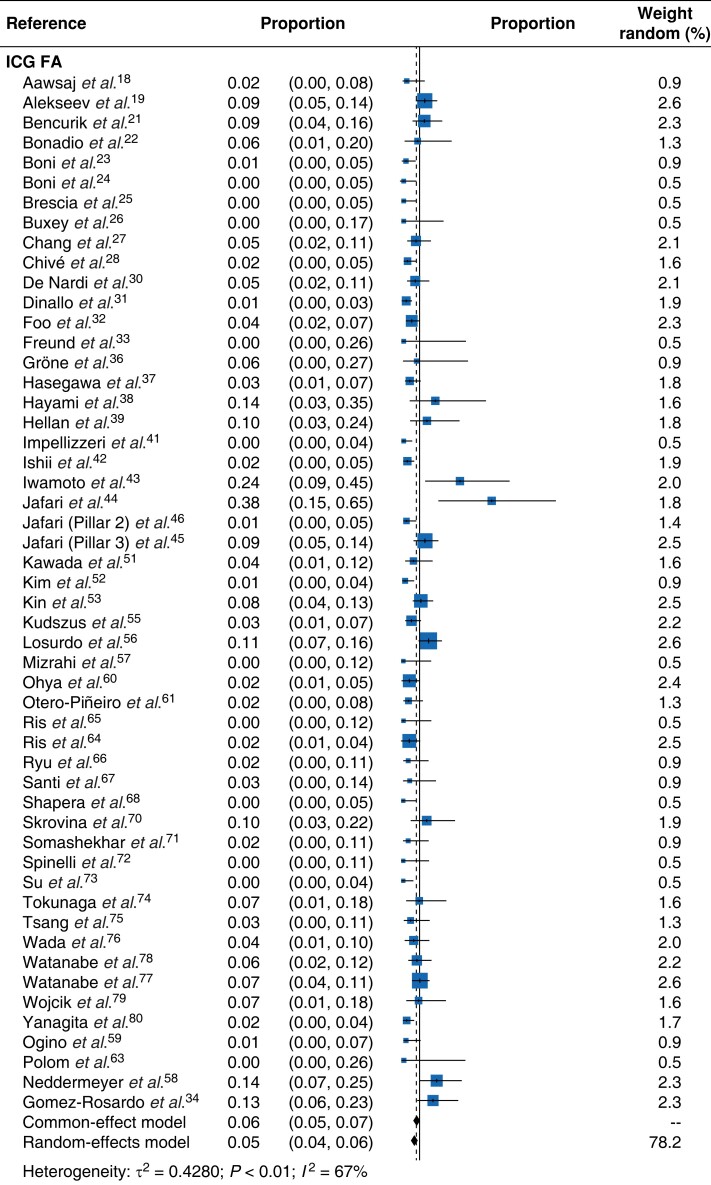

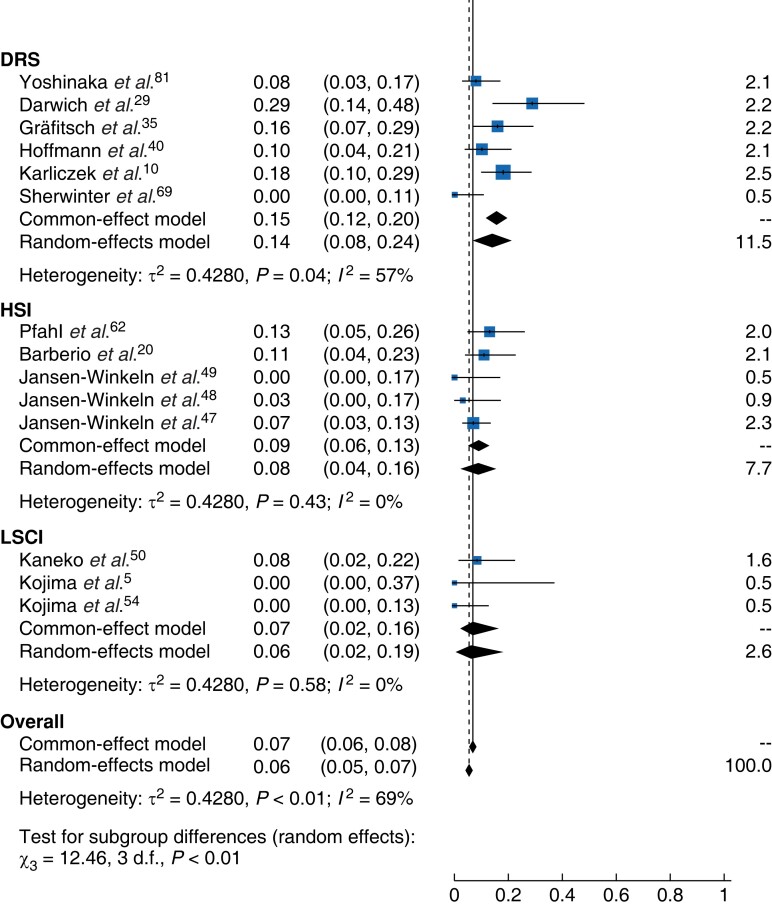
Forest plot for each perfusion assessment method and risk of anastomotic leak Proportions are shown with 95 per cent confidence intervals. ICG-FA, indocyanine green fluorescence angiography; DRS, diffuse reflectance spectroscopy; HSI, hyperspectral imaging; LSCI, laser speckle contrast imaging.

Detailed results of the meta-analysis are presented in a form of forest plot (*Fig. 2*). The pooled effects of anastomotic leak per case derived from the random-effects model was 0.05 (95 per cent c.i. 0.04 to 0.07). The effect derived from the common-effect model was very similar at 0.07 (0.06, 0.08). The estimated effects ranged from 0 to 0.38^44^. There was heterogeneity in the data (*I*^2^ = 69 per cent, τ^2^ = 0.5335, χ^2^ = 211.70, 65 d.f., *P* < 0.01). *Figure 3* illustrates the effect each perfusion assessment modality on anastomotic leak rates. The effects of different modalities were estimated using a random-effects model with a modality factor included as categorical predictor. Different modalities in this equation were represented by dummy variables, with ICG-FA fixed as a reference category. The results of this analysis indicated that 19.8 per cent of the overall heterogeneity of studies related to the different perfusion assessment modalities. The test of residual heterogeneity indicated that, after accounting for different modalities, there remained significant heterogeneity in the effects caused by other factors (QE (62 d.f.) = 172.8238, *P* < 0.001). All effects were significantly different from zero, indicating that the modalities tested reduced the risk of an adverse outcome (*Table 3*) which in this instance was anastomotic leak. The detailed subgroup effects, along with their 95 per cent confidence intervals and heterogeneity estimate, are presented in *Table 3*. The omnibus test for the equality of effects for different modalities indicated that they also differed significantly between each other (QM (4 d.f.) = 646.1490, *P* < 0.001). Further tests revealed that significant differences in results existed between ICG-FA and DRS, as well as between pairs HSI and DRS, and LSCI and DRS. The estimated effect for the pooled cohort of all control cases, where no perfusion assessment was used, was 0.10 (0.08, 0.12).

**Table 3:** Results of meta-analysis investigating risk of anastomotic leak by perfusion assessment modality and overall

Modality	AL estimates taken from random-effects model*	Heterogeneity				Weight (%)
		*I* ^2^ (%)	τ^2^	χ^2^	*P*	
**All modalities**	0.06 (0.05, 0.07)	69	0.4280	$\chi_{65}^{2}\;$ = 211.70	<0.01	100
ICG-FA	0.05 (0.04, 0.06)	67	0.4280	$\chi_{51}^{2}\;$ = 156.37	<0.01	78.2
DRS	0.14 (0.08, 0.24)	57	0.4280	$\chi_{5}^{2}\;$ = 11.55	0.04	11.5
HSI	0.08 (0.04, 0.16)	0	0.4280	$\chi_{4}^{2}\;$ = 3.8	0.43	7.7
LSCI	0.06 (0.02, 0.19)	0	0.4280	$\chi_{2}^{2}\;$ = 1.09	0.58	2.6
Control	0.10 (0.08, 0.12)	80	0.3464	$\chi_{32}^{2}\;$ = 162.52	<0.01	100

Values in parentheses are 95% confidence intervals. Amount of total heterogeneity accounted for by modalities (R^2^) = 19.8%. AL, anastomotic leak; ICG-FA, indocyanine green fluorescence angiography; DRS, diffuse reflectance spectroscopy; HSI, hyperspectral imaging; LSCI, laser speckle contrast imaging.

Because the data revealed a substantial level of heterogeneity, further procedures were employed, to search for possible explanations for the observed differences in effect sizes between studies. A subgroup analysis was used for this purpose. The main focus of the subgroup analysis was performance modality. The effects of other potential moderators were not a subject of interest here as a number of other meta-analyses are available in the literature^87–91^. The summary effect sizes for each modality (ICG-FA, DRS, HSI, LSCI) were calculated and reported in a separate forest plot (*Fig. 3*). The final part of the statistical analysis was to investigate possible publication bias; the funnel plot was symmetrical (*supplementary material*)^15^.

### Quality assessment

**Fig. 4:**
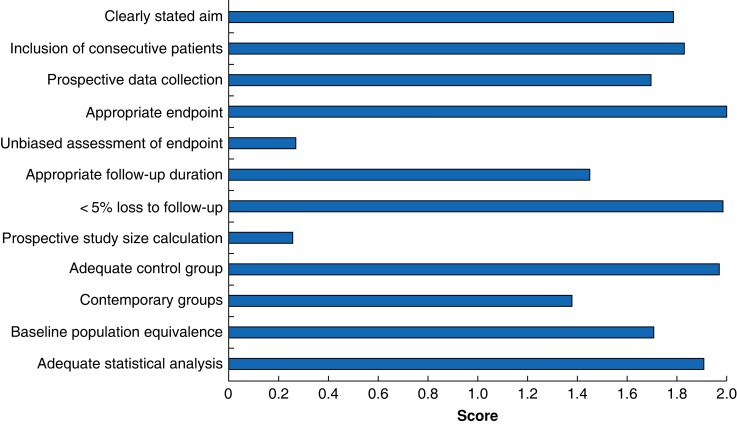
Average (mean) MINORS scores for all studies The last four categories were only applicable to controlled trials. Maximum scores per category were 2 and minimum scores available were 0. MINORS, Methodological Index for Non-Randomized Studies.

The average MINORS score for controlled trials was 18 (range 13–24) of 24. For non-controlled trials it was 11 (8–14) of 16. Most studies lost points for lack of blinding, or for not calculating study size or powers, and so were deemed to have a high risk of bias in these domains. The breakdown for individual categories is shown in *Fig. 4*. The greater the score for each domain, the less susceptible it is to bias. Full MINORS scores for each paper are documented in the *supplementary material*.

## Discussion

This meta-analysis found that assessment of bowel perfusion before the formation of a colorectal anastomosis reduced the incidence of anastomotic leak. ICG-FA, DRS, LSCI, and HSI all reduced the risk of anastomotic leak occurring for the included populations.

This comprehensive review compared four methods of bowel perfusion assessment. The major limitation of the study pertained to the limited number of studies and case populations in all groups other than ICG-FA, meaning that studies describing other imaging modalities were potentially underpowered. With ICG-FA accounting for 78.2 per cent of all cases, the total anastomotic leak rate is likely to be skewed more towards the mean for ICG-FA, rather than the true mean across all studies. Additionally, MINORS scores for the included studies were low for blinding and study sizes. This is likely to have influenced the results in favour of the interventions investigated. Owing to the heterogeneity of the results, it was decided to rely on results obtained from random-effects model methodology as it incorporates more realistic assumptions about heterogeneity of effects across studies. The data sets from the DRS and ICG-FA groups demonstrated the most heterogeneity. Factors increasing study heterogeneity included different study methodologies, definitions of anastomotic leak, surgical techniques, and device use. Studies investigating HSI and LSCI had lower heterogeneity as they were trials of similar origin.

The current literature is conflicting regarding the efficacy of ICG-FA. Similar to other work, based on the present review, the authors propose that ICG-FA has a role in reducing the risk of anastomotic leak when deployed during operation for lower gastrointestinal cancers^61,86,88,92^. However, some of the larger case–control studies evaluating ICG-FA drew contrasting conclusions; some^42,45^ suggested a trend towards reduced anastomotic leak rates with the use of ICG-FA, whereas others^19,32,77^ demonstrated that ICG-FA could significantly reduce leakage rates. Of note, there are also a number of ongoing studies assessing the benefits of ICG-FA in reducing anastomotic leak rates in colorectal cancers, including EssentiAL^93^, IntAct, and AVOID.

The total random-effects results demonstrated that DRS was not as effective as HSI, LSCI, and ICG-FA in reducing the risk of anastomotic leak. This is also supported by the differences between DRS and the other imaging modalities in omnibus testing. A potential reason for this is that DRS looks at a very small area of tissue oxygenation and so lacks the overall picture that ICG-FA, HSI or LSCI may provide; further research to test this hypothesis is recommended.

At present, there is no consensus regarding the definition of reduced perfusion across all modalities. For ICG-FA, a visual marker of perfusion is generated and studies^31,53,56,60,68^ have used various uptake time cut-offs from 25 to 60 s, and differing volumes of ICG, to define optimal perfusion. However, this relies on an effective systemic vascular supply. There is also variation in the literature concerning the range of healthy tissue saturation levels. Mean colonic *S*to_2_ measurements from the DRS group indicated that a reduced risk of anastomotic leak was associated with a value of between 58 and 79.4 per cent, and that the lowest *S*to_2_ measurement for a viable anastomosis was 51 per cent^10,35,40^. Additionally, in the DRS group, it was proposed that an *S*to_2_ rise of 2 per cent after anastomosis formation had to occur to avoid leakage^10,38^. The wide range of proposed healthy tissue saturation may be explained by the specific equipment used, with certain systems having lower cut-offs.

The lack of objective measurement with ICG-FA is an active area of research. One of the included studies considered the development of quantitative fluorescence measurement within ICG-FA, with the aim of measuring the fluorescence of ICG objectively and relaying it back to the surgeon for a strengthened anastomotic line. They proposed an arbitrary unit cut-off but were limited by hypertension and location of the anastomosis, as well as a lack of real-time evaluation as data were processed after operation^34^. The development of a quantitative cut-off for adequate perfusion and its validation during surgery would likely enhance its use and uptake.

There also remain drawbacks in implementing the other imaging methods investigated. DRS uses a probe-based measurement of serosal oxygenation, where only a small amount of tissue is measured; LSCI requires a separate camera system to view data that can be used for surgical visualization; and, at present, HSI has a near-to but not real-time laparoscopic system^8^. In the fields of HSI and LSCI, studies are being set up to assess whether wide-field imaging for perfusion measurements and concurrent tissue differentiation can reliably be performed in real time^94,95^.

Finally, none of the included perfusion assessment methods currently have validated protocols documented in the reviewed literature and no standard perfusion assessment modality exists for colorectal resection. LSCI, HSI, and DRS are emerging technologies, as evidenced by the smaller number of included studies and limited numbers of patients. Future work across all modalities will require the development of standardized protocols for ease of adoption and use. The use of adjunctive bowel perfusion measurement technologies is unlikely to negate the importance of surgical skill and experience, but rather should promote safer surgery during bowel resections, and surgical centres wishing to adopt new technology should take these factors into consideration.

## Supplementary Material

znad154_Supplementary_Data

## Data Availability

The full data set is available to view in the *supplementary material*. Should further information or data be required, the corresponding author can be contacted.
