# Supplementary material for: Intraoperative bowel perfusion assessment methods and their effects on anastomotic leak rates: meta-analysis
Source: Br J Surg. 2023 May 30;110(9):1131–42. doi: 10.1093/bjs/znad154 (PMC10416696; doi:10.1093/bjs/znad154)
Supplement: znad154_Supplementary_Data [file znad154_supplementary_data.zip › Supplementary_Material.docx]

**Intraoperative bowel perfusion assessment methods and their effects on anastomotic leak rates: meta-analysis**

Maxwell S Renna^1,2^, Mariusz T. Grzeda^1^, James Bailey^3^, Alison Hainsworth^2^, Sebastien Ourselin^1,4^, Michael Ebner^4^, Tom Vercauteren^1,4^, Alexis Schizas^2^, Jonathan Shapey^1,4,5^

^1^ King’s College London, ^2^ Guy’s & St. Thomas’ NHS Foundation Trust, London, ^3^ University of Nottingham, ^4^Hypervision Surgical Ltd, ^5^ King’s College Hospital, London

Maxwell S Renna – [max.renna@kcl.ac.uk](mailto:max.renna@kcl.ac.uk), 9^th^ Floor Beckett House, 1 Lambeth Palace Rd, South Bank, London, SE1 7EU, ORCID ID: 0000-0002-0136-4311, Twitter: _MaxwellSR

**Supplementary Materials - Index**

| **Supplementary Methods** |  |
| --- | --- |
| Exclusion reasons | *pag. 2* |
| MINORS Criteria and Scoring Results | *pag. 2* |
| **Supplementary Results** |  |
| Funnel Plot | *pag. 5* |
| Dataset | *pag. 5* |
|  |  |
|  |  |
|  |  |
|  |  |
|  |  |
|  |  |
|  |  |
|  |  |

**Supplementary Methods**

## Exclusion reasons:

| **Exclusion Reason** | **Count** |
| --- | --- |
| Exclude - abstract only | 52 |
| Exclude - book section | 1 |
| Exclude - case report/series/study | 104 |
| Exclude - Not English | 2 |
| Exclude – Incorrect study type | 5 |
| Exclude - focusses on type of surgery not imaging modality | 1 |
| Exclude - gynae cancer | 1 |
| Exclude - inaccessible | 7 |
| Exclude - No Anastomotic leak data | 4 |
| Exclude - no demographic data | 2 |
| Exclude - no intraoperative imaging/perfusion data | 3 |
| Exclude – non-clinical study | 2 |
| Exclude – non-human | 10 |
| Exclude - not relevant | 28 |
| Exclude - opinion | 3 |
| Exclude - paediatrics | 2 |
| Exclude - porcine | 1 |
| Exclude - preop imaging only | 1 |
| Exclude - review article | 4 |
| Exclude - transplant | 2 |
| Exclude - Upper GI | 3 |
| Exclude - systematic review | 17 |

## MINORS Criteria and Scoring Results(87)

**1. A clearly stated aim: the question addressed should be precise and relevant in the light of available literature**

0 = not included or commented on

1 = Specifically mentions intervention or outcome

2 = Specifically mentions intervention and outcome for a specific procedure

**2. Inclusion of consecutive patients: all patients potentially fit for inclusion (satisfying the criteria for inclusion) have been included in the study during the study period (no exclusion or details about the reasons for exclusion)**

0 = Not included or commented on

1 = Unclear exclusion/inclusion criteria

2 = Inclusion/exclusion criteria reported

**3. Prospective collection of data: data were collected according to a protocol established before the beginning of the study**

0 = Not disclosed

1 = Not prospective

2 = Prospective

**4. Endpoints appropriate to the aim of the study: unambiguous explanation of the criteria used to evaluate the main outcome which should be in accordance with the question addressed by the study. Also, the endpoints should be assessed on an intention-to-treat basis.**

0 = Not included or commented on

1 = Endpoint not appropriate to aims

2 = Appropriate Endpoint

**5. Unbiased assessment of the study endpoint: blind evaluation of objective endpoints and double-blind evaluation of subjective endpoints. Otherwise the reasons for not blinding should be stated**

0 = Not included or commented on

1 = Reason for not blinding stated but study not blinded/data inputted anonymously

2 = Blinded

**6. Follow-up period appropriate to the aim of the study: the follow-up should be sufficiently long to allow the assessment of the main endpoint and possible adverse events**

0 = Not included or commented on

1 = Follow-up less than 30 days or follow-up duration not disclosed but morbidity or mortality included

2 = Follow up 30 days or more

**7. Loss to follow up less than 5%: all patients should be included in the follow up. Otherwise, the proportion lost to follow up should not exceed the proportion experiencing the major endpoint**

0 = Not included or commented on

1 = More than 5% lost to follow-up

2 = Less than 5% lost to follow-up

**8. Prospective calculation of the study size: information of the size of detectable difference of interest with a calculation of 95% confidence interval, according to the expected incidence of the outcome event, and information about the level for statistical significance and 2 estimates of power when comparing the outcomes Additional criteria in the case of comparative study**

0 = Not included or commented on or insufficient sample size

1 = Prospective calculation without power analysis

2 = Prospective calculation with power analysis

**9. An adequate control group: having a gold standard diagnostic test or therapeutic intervention recognized as the optimal intervention according to the available published data**

0 = Not included or commented on

1 = Not applicable

2 = Control with no imaging or other method of imaging if comparative study (as no gold standard exists)

**10. Contemporary groups: control and studied group should be managed during the same time period (no historical comparison)**

0 = Not included or commented on

1 = Historical controls

2 = Prospective controls

**11. Baseline equivalence of groups: the groups should be similar regarding the criteria other than the studied endpoints. Absence of confounding factors that could bias the interpretation of the results**

0 = Not included or commented on

1 = Baseline characteristics not equivalent but included or not compared statistically

2 = Matched characteristics or similar characteristics

**12. Adequate statistical analyses: whether the statistics were in accordance with the type of study with calculation of confidence intervals or relative risk**

0 = Not included or commented on

1 = Analysis present but no description or analysis

2 = Adequate analysis with description and reasoning

**The items are scored 0 (not reported), 1 (reported but inadequate) or 2 (reported and adequate). The global ideal score is 16 for non-comparative studies and 24 for comparative studies.**

| Author | 1 | 2 | 3 | 4 | 5 | 6 | 7 | 8 | 9 | 10 | 11 | 12 | Total | Max score available |
| --- | --- | --- | --- | --- | --- | --- | --- | --- | --- | --- | --- | --- | --- | --- |
| Aawsaj 2021 | 2 | 1 | 0 | 2 | 2 | 0 | 2 | 0 | 1 | 0 | 2 | 1 | 13 | 24 |
| Alekseev 2020 | 2 | 2 | 2 | 2 | 1 | 2 | 2 | 2 | 2 | 2 | 2 | 2 | 23 | 24 |
| Bencurik 2017 | 2 | 2 | 2 | 2 | 1 | 1 | 1 | 0 | 2 | 2 | 2 | 2 | 19 | 24 |
| Bonadio 2020 | 2 | 1 | 0 | 2 | 0 | 2 | 2 | 0 | 2 | 1 | 1 | 2 | 15 | 24 |
| Boni 2016 | 1 | 2 | 1 | 2 | 0 | 0 | 2 | 0 | - | - | - | - | 8 | 16 |
| Boni 2017 | 2 | 2 | 2 | 2 | 0 | 1 | 2 | 0 | 2 | 1 | 1 | 2 | 17 | 24 |
| Brescia 2018 | 1 | 0 | 0 | 2 | 0 | 1 | 2 | 0 | 2 | 1 | 2 | 2 | 13 | 24 |
| Buxey 2019 | 1 | 2 | 2 | 2 | 0 | 1 | 2 | 0 | - | - | - | - | 10 | 16 |
| Chang 2019 | 2 | 2 | 2 | 2 | 2 | 2 | 2 | 0 | - | - | - | - | 14 | 16 |
| Chive 2021 | 2 | 1 | 2 | 2 | 0 | 2 | 2 | 0 | 2 | 1 | 1 | 2 | 17 | 24 |
| Darwich 2019 | 2 | 2 | 2 | 2 | 0 | 1 | 2 | 0 | 2 | 2 | 2 | 2 | 19 | 24 |
| De Nardi 2020 | 2 | 2 | 2 | 2 | 2 | 2 | 2 | 2 | 2 | 2 | 2 | 2 | 24 | 24 |
| Dinallo 2019 | 2 | 1 | 1 | 2 | 0 | 0 | 2 | 0 | 2 | 1 | 1 | 1 | 13 | 24 |
| Foo 2020 | 2 | 2 | 1 | 2 | 0 | 2 | 2 | 0 | 2 | 1 | 2 | 2 | 18 | 24 |
| Freund 2021 | 2 | 2 | 2 | 2 | 0 | 1 | 2 | 0 | 2 | 2 | 2 | 2 | 19 | 24 |
| Grafitsch 2021 | 2 | 2 | 2 | 2 | 1 | 1 | 2 | 0 | 2 | 2 | 2 | 2 | 20 | 24 |
| Groene 2015 | 2 | 2 | 2 | 2 | 0 | 2 | 2 | 0 | - | - | - | - | 12 | 16 |
| Hasegawa 2020a | 2 | 2 | 2 | 2 | 0 | 2 | 2 | 0 | 2 | 1 | 2 | 2 | 19 | 24 |
| Hayami 2019 | 2 | 2 | 2 | 2 | 0 | 1 | 2 | 0 | - | - | - | - | 11 | 16 |
| Hellan 2014 | 2 | 2 | 2 | 2 | 2 | 2 | 2 | 0 | - | - | - | - | 14 | 16 |
| Hoffmann 2017 | 2 | 2 | 2 | 2 | 0 | 2 | 2 | 2 | - | - | - | - | 14 | 16 |
| Impellizzeri 2020 | 2 | 2 | 1 | 2 | 0 | 2 | 2 | 0 | 2 | 1 | 1 | 2 | 17 | 24 |
| Ishii 2020 | 1 | 2 | 1 | 2 | 0 | 2 | 2 | 0 | 2 | 2 | 1 | 2 | 17 | 24 |
| Iwamoto 2020 | 2 | 2 | 2 | 2 | 0 | 2 | 2 | 0 | - | - | - | - | 12 | 16 |
| Jafari 2013 | 2 | 2 | 1 | 2 | 0 | 2 | 2 | 0 | 2 | 2 | 1 | 2 | 18 | 24 |
| Jafari 2015 Pillar 2 | 2 | 2 | 2 | 2 | 0 | 2 | 2 | 0 | - | - | - | - | 12 | 16 |
| Jafari 2021 Pillar 3 | 2 | 2 | 2 | 2 | 1 | 2 | 2 | 2 | 2 | 2 | 1 | 2 | 22 | 24 |
| Jansen-Winkeln 2019 | 2 | 2 | 2 | 2 | 0 | 2 | 2 | 0 | - | - | - | - | 12 | 16 |
| Kaneko 2020 | 1 | 2 | 2 | 2 | 0 | 2 | 2 | 0 | 2 | 2 | 2 | 2 | 19 | 24 |
| Karliczek 2010 | 1 | 2 | 2 | 2 | 0 | 2 | 2 | 0 | - | - | - | - | 11 | 16 |
| Kawada 2017 | 2 | 2 | 2 | 2 | 2 | 2 | 2 | 0 | - | - | - | - | 14 | 16 |
| Kim 2016 | 2 | 2 | 2 | 2 | 0 | 2 | 2 | 0 | 2 | 1 | 1 | 2 | 18 | 24 |
| Kin 2015 | 2 | 2 | 1 | 2 | 0 | 1 | 2 | 1 | 2 | 1 | 2 | 2 | 18 | 24 |
| Kojima 2019 | 2 | 2 | 2 | 2 | 0 | 2 | 2 | 2 | - | - | - | - | 14 | 16 |
| Kojima 2020 | 2 | 2 | 2 | 2 | 0 | 2 | 2 | 0 | 2 | 1 | 2 | 2 | 19 | 24 |
| Kudszus 2010 | 2 | 1 | 1 | 2 | 0 | 1 | 2 | 0 | 2 | 1 | 1 | 2 | 15 | 24 |
| Losurdo 2020 | 2 | 2 | 1 | 2 | 0 | 1 | 2 | 0 | 2 | 1 | 2 | 2 | 17 | 24 |
| Mizrahi 2018 | 2 | 2 | 2 | 2 | 0 | 1 | 2 | 0 | 2 | 1 | 2 | 2 | 18 | 24 |
| Ogino 2018 | 1 | 2 | 2 | 2 | 0 | 2 | 2 | 0 | - | - | - | - | 11 | 16 |
| Ohya 2020 | 2 | 2 | 1 | 2 | 0 | 1 | 2 | 0 | - | - | - | - | 10 | 16 |
| Otero-Pineiro 2021 | 2 | 2 | 2 | 2 | 1 | 2 | 2 | 0 | 2 | 1 | 2 | 2 | 20 | 24 |
| Ris 2014 | 1 | 1 | 2 | 2 | 0 | 2 | 2 | 0 | - | - | - | - | 10 | 16 |
| Ris 2018 | 2 | 2 | 2 | 2 | 1 | 2 | 2 | 1 | - | - | - | - | 14 | 16 |
| Ryu 2020a | 1 | 2 | 2 | 2 | 0 | 0 | 2 | 0 | - | - | - | - | 9 | 16 |
| Santi 2018 | 2 | 2 | 2 | 2 | 0 | 2 | 2 | 0 | - | - | - | - | 12 | 16 |
| Shapera 2019 | 0 | 2 | 2 | 2 | 0 | 1 | 2 | 0 | - | - | - | - | 9 | 16 |
| Sherwinter 2021 | 1 | 2 | 2 | 2 | 0 | 1 | 2 | 0 | - | - | - | - | 10 | 16 |
| Skrovina 2020 | 2 | 2 | 1 | 2 | 0 | 1 | 2 | 0 | 2 | 1 | 2 | 1 | 16 | 24 |
| Somashekhar 2020 | 1 | 2 | 2 | 2 | 0 | 1 | 2 | 0 | - | - | - | - | 10 | 16 |
| Spinelli 2019 | 2 | 2 | 1 | 2 | 0 | 1 | 2 | 0 | 2 | 1 | 2 | 2 | 17 | 24 |
| Su 2020 | 2 | 2 | 2 | 2 | 0 | 2 | 2 | 0 | 2 | 2 | 2 | 2 | 20 | 24 |
| Tokunaga 2021 | 2 | 2 | 2 | 2 | 0 | 1 | 2 | 0 | - | - | - | - | 11 | 16 |
| Tsang 2020 | 2 | 2 | 2 | 2 | 0 | 2 | 2 | 0 | 2 | 2 | 2 | 2 | 20 | 24 |
| Wada 2017 | 2 | 2 | 1 | 2 | 0 | 1 | 2 | 0 | - | - | - | - | 10 | 16 |
| Watanabe 2015 | 2 | 2 | 2 | 2 | 0 | 1 | 2 | 0 | - | - | - | - | 11 | 16 |
| Watanabe 2020 | 2 | 2 | 2 | 2 | 0 | 2 | 2 | 2 | 2 | 2 | 2 | 2 | 22 | 24 |
| Wojcik 2020 | 2 | 2 | 2 | 2 | 1 | 1 | 2 | 0 | 2 | 2 | 2 | 2 | 20 | 24 |
| Yanagita 2021 | 1 | 2 | 2 | 2 | 0 | 1 | 2 | 2 | 2 | 1 | 2 | 2 | 19 | 24 |
| Jansen-Winkeln 2021 | 2 | 2 | 2 | 2 | 0 | 1 | 2 | 0 | - | - | - | - | 11 | 16 |
| Jansen-Winkeln 2022 | 2 | 2 | 2 | 2 | 1 | 2 | 2 | 0 | - | - | - | - | 13 | 16 |
| Yoshinaka 2022 | 2 | 2 | 2 | 2 | 0 | 1 | 2 | 0 | - | - | - | - | 11 | 16 |
| Polom 2022 | 2 | 1 | 2 | 2 | 0 | 1 | 2 | 0 | - | - | - | - | 10 | 16 |
| Pfahl 2022 | 2 | 2 | 2 | 2 | 0 | 2 | 2 | 0 |  |  |  |  | 12 | 16 |
| Neddermeyer 2022 | 2 | 1 | 1 | 2 | 0 | 2 | 2 | 1 | 2 | 1 | 2 | 2 | 18 | 24 |
| Gomez Rosardo 2022 | 2 | 1 | 2 | 2 | 0 | 1 | 2 | 0 | - | - | - | - | 10 | 16 |
| Barberio 2022 | 2 | 2 | 2 | 2 | 0 | 1 | 2 | 0 | - | - | - | - | 11 | 16 |

**Supplementary Results:**

## Funnel plot

##
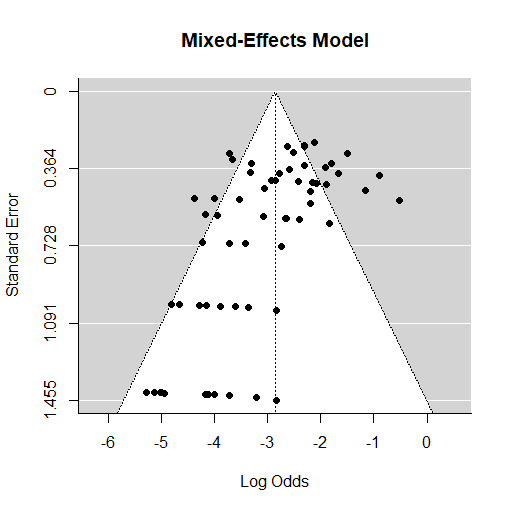


## Dataset

| **Author** | **Year** | **Country** | **Study Design** | **Study centres** | **Number of Participants** | **Included numbers** | | **Age** | | **Sex** | | | | **BMI** | | **Procedure focus** | **Imaging system** | **ICG/Imaging complications** | **Operation Time** | | **Intraop perfusion modality** | **Site relocation data** | **Al rates (<30 days)** | | | **Reoperation** | | **AL definition** | |
| --- | --- | --- | --- | --- | --- | --- | --- | --- | --- | --- | --- | --- | --- | --- | --- | --- | --- | --- | --- | --- | --- | --- | --- | --- | --- | --- | --- | --- | --- |
|  |  |  |  |  | **Total** | **Case** | **Control** | **Case** | **Cont** | **Case Male** | **Case Female** | **Cont male** | **Cont Female** | **Case** | **Control** |  |  |  | **Case** | **Control** |  |  | **Case** | **Control** | **Case** | | **Control** |  |  |
| Aawsaj | 2021 | UK | Retrospective case-control | Single centre | 127 | 65 | 62 | 69.5 | 68.9 | 22 | 43 | 32 | 30 | 27.6 | 28.3 | Right sided colonic resection | Pulsion Medical Systems, | 0 | 164.7 | 162.6 | ICG FA | 4 | 1 | 3 | 3 | | 2 | Not included |  |
| Alekseev | 2020 | Russia | Randomised Control Trial | Single centre | 377 | 187 | 190 | 63 | 63 | 92 | 95 | 92 | 98 | NA | NA | LAR, low anterior rectal resection; LC, left colectomy, AR, anterior resection; | KARL STORZ GmbH &Co. KG, Tuttlingen, Germany) with a light source (D-LIGHT P SCB; KARL STORZ) | 0 | 176 | 185 | ICG FA | 36 | 17 | 31 | 7 | | 4 | International Study Group of Rectal Cancer |  |
| Bencurik | 2017 | Czech Republic | Case Control | Single centre | 200 | 100 | 100 | 62.6 | 64.6 | 66 | 34 | 64 | 36 | 26.8 | 27.3 | TME for rectal Cancer | SPIES system (KARL STORZ GmbH & Co. KG, Tuttlingen, Germany) or Firefly™ robotic surgical system da Vinci Xi ® (Intuitive Surgical, Sunnyvale, CA, USA). | 0 | 259 | 226 | ICG FA | 15 | 9 | 19 | 14 | | 11 | International Study Group of Rectal Cancer |  |
| Bonadio | 2019 | Italy | Retrospective Cohort Analysis | Single centre | 66 | 33 | 33 | 71.85 | 69.03 | 21 | 12 | 15 | 18 | 25.6 | 25.7 | Rectal anterior resection for extraperitoneal cancer | SPIES system (KARL STORZ GmbH&Co. KG, Tuttlingen, Germany) and a full high-definition camera system (IMAGE 1 SPIESTM, KARL STORZ). A xenon light source was employed (D-LIGHT P SCB, KARL STORZ) | 0 | 217 | 201 | ICG FA | 6 | 2 | 7 | NA | | NA | International Study Group on Rectal Cancer |  |
| Boni | 2016 | Italy | Interventional Studies Without Concurrent Controls | Single centre | 107 | 107 | 0 | 69 | NA | 60 | 47 | NA | NA | 25 | NA | Lap colorectal resection inc R + L colectomy, ant resection + splenic flexure resections | (KARL STORZ GmbH & Co. KG, Tuttlingen, Germany) (IMAGE 1 SPIESTM, KARL STORS) STORZ) | 0 | 130 | NA | ICG FA | 4 | 1 | NA | 1 | | NA | Clavien/Dindo |  |
| Boni | 2017 | Italy | Interventional Studies Without Concurrent Controls | Single centre | 136 | 69 | 67 | 69 | 68 | 28 | 14 | 22 | 16 | 27 | 29 | laparoscopic anterior resection with total mesorectal excision (TME). | (KARL STORZ GmbH & Co. KG, Tuttlingen, Germany) (IMAGE 1 SPIESTM, KARL STORS) STORZ) | 0 | 165 | 172 | ICG FA | 2 | 0 | 2 | 0 | | 1 | Clavien/Dindo |  |
| Brescia | 2018 | Italy | Retrospective Case–Control Study | Single centre | 182 | 75 | 107 | 67.1 | 65.7 | 43 | 32 | 63 | 44 | 24.4 | 25.6 | L + R colectomy + ant resection | (IMAGE 1 SPIES™ system, KARL STORZ GmbH & Co.KG, Tuttlingen, Germany) (KARL STORZ GmbH & Co.KG, Tuttlingen, Germany) | 0 | 180 | 175 | ICG FA | 5 | 0 | 6 | 0 | | 2 | Dindo–Clavien grades II and III) |  |
| Buxey | 2019 | Australia | Interventional Studies Without Concurrent Controls | Single centre | 20 | 20 | 0 | 65.55 | NA | 11 | 9 | NA | NA | NA | NA | L hemicolectomy, ant resections, total colectomy | NA | 0 | NA | NA | ICG FA | 0 | 0 | NA | NA | | NA | Not defined |  |
| Chang | 2019 | China | Interventional Studies Without Concurrent Controls | Single centre | 110 | 110 | 0 | 67 | NA | 71 | 39 | NA | NA | 23.7 | NA | Left sided colorectal resection | NA | NA | 232 | NA | ICG FA | 34 | 6 | NA | NA | | NA | Not defined |  |
| Chive | 2021 | France | Retrospective, Propensity Score Matching Study Case Control | Single centre | 835 | 158 | 677 | 64 | 62 | 95 | 63 | 374 | 303 | 26.1 | 26.5 | L + R + ant resection | (a Photodynamic Eye PC6100 C9830−10 from Hamamatsu, Hamamatsu, Japan, or a Novadaq from Stryker, Kalamazoo, MI) | NA | NA | NA | ICG FA | 6 | 3 | 39 | NA | | NA | The presence of AL was suggested by clinical signs of peritonitis or a serum C-reactive protein level above 150 mg/l on POD 3. The leakage was then confirmed by the computed tomography (CT) scan results with a water-soluble contrast enema, i.e., extraluminal contrast, clear extravasation from the suture line, or (in the absence of extraluminal contrast) an abscess close to the anastomosis. |  |
| Darwich | 2019 | Germany | Interventional Studies Without Concurrent Controls | Single centre | 31 | 31 | 0 | 66.74 | NA | 15 | 16 | NA | NA | NA | NA | LAR for rectal Ca | “Oxygen to See” (O2C®, LEA-Medizintechnik, Giessen, germany) | Probe broke in 3 patients | NA | NA | STO2 | NA | 9 | NA | NA | | NA | ISREC |  |
| De Nardi | 2020 | Italy | Randomised Control Trial | Multi-centre | 240 | 118 | 122 | 66.1 | 65.1 | 60 | 58 | 66 | 56 | 25.2 | 25.6 | Left colectomy and LAR | KARL STORZ GmbH & Co. KG, Tuttlingen, Germany) | 0 | 192.5 | 187.5 | ICG FA | 11 | 6 | 11 | NA | | NA | ISREC |  |
| Dinallo | 2019 | USA | Retrospective Case-Control Analysis | Single Centre | 554 | 320 | 234 | 61.5 | 62.5 | 108 | 126 | 138 | 182 | 28.3 | 28.3 | R hemi, tranverse colectomy, LAR, left hemi, subtotal, total proctocolectomy, hartmann's reversal | SPY elite | 0 | 234.9 | 214.5 | ICG FA | 8.5 | 4 | 3 | NA | | NA | NA |  |
| Foo | 2020 | Hong Kong | Retrospective Case Control | Single centre | 506 | 253 | 253 | 66.6 | 67.2 | 166 | 87 | 163 | 90 | NA | NA | Anterior + LAR | SPY Elite System, the Pinpoint System and he Da Vinci Xi Firefy system | NA | 218.2 | 227 | ICG FA | 53 | 0 | 0 | 1.6 | | 2 | ISREC |  |
| Freund | 2021 | USA | Retrospective Case-control | Single centre | 36 | 12 | 24 | 53.5 | 58 | 5 | 7 | 13 | 11 | 24.1 | 35.6 | Redo ileocolic resections | PINPOINT Endoscips system (Stryker) | NA | 255 | 255.5 | ICG FA | NA | 0 | 1 | NA | | NA | Not defined |  |
| Grafitsch | 2021 | Switzerland | Interventional Studies Without Concurrent Controls | Single centre | 50 | 50 | 0 | 66 | NA | 34 | 16 | NA | NA | 23.6 | NA | Left sided colorectal resections | VLS probe (T-Stat®, Ischemia Detection System, Spectros Corp., pOTOLA vALLET, ca, usa) | NA | 198 | NA | STO2 | NA | 8 | NA | NA | | NA | NA |  |
| Groene | 2015 | Germany | Interventional Studies Without Concurrent Controls | Single centre | 18 | 18 | 0 | 66 | NA | 8 | 10 | NA | NA | NA | NA | Lap low rectal/anorectal anastomoses | PINPOINT Endoscopic Fluorescence Imaging Sys- | NA | NA | NA | ICG FA | 5 | 1 | NA | 1 | | NA | not defined |  |
| Hasegawa | 2020 | Japan | Retrospective Cohort Analysis | Single centre | 844 | 141 | 703 | 63 | 62 | 99 | 42 | 450 | 253 | 22.3 | 22.9 | Lap low anterior resections or intersphincteric resection for malignant rectal tumours | the IMAGE1 STM system (Karl Storz SE & Co. KG, Tuttlingen, Germany), 1588 Advanced Imaging Modalities System Handy (Mizuho Medical Co. Ltd., Tokyo, Japan).(Stryker, Kalamazoo, MI, USA), or HyperEye Medical (AIM) Platform and SPY Fluorescence technology | 0 | NA | NA | ICG FA | 24 | 4 | 87 | NA | | NA | AL was implied by the presence of clinical symptoms such as the discharge of gas or feces from the pelvic drain or wound, or fistula formation, as previously described |  |
| Hayami | 2019 | Japan | Interventional Studies Without Concurrent Controls | Single centre | 22 | 22 | 0 | 69 | NA | 7 | 15 | NA | NA | 22.8 | NA | Lap surgery for colorectal Cancer | (D-light P system, Karl Storz, Tuttlingen, Germany). | 0 | NA | NA | ICG FA | NA | 3 | NA | NA | | NA | not defined |  |
| Hellan | 2014 | USA | Interventional Studies Without Concurrent Controls | Multi-centre | 40 | 40 | 0 | 63.9 | NA | 20 | 20 | NA | NA | 27.6 | NA | robotic left sided colorectal surgery | The fluorescence-capable da Vinci Si high-definition (HD) vision system (Firefly) | 0 | 232 | NA | ICG FA | 16 | 4 | NA | 4 | | NA | not defined |  |
| Hoffmann | 2017 | Switzerland | Interventional Studies Without Concurrent Controls | Single centre | 58 | 58 | 0 | 71 | NA | 37 | 21 | NA | NA | 24 | NA | Colorectal resections | (T-Stat, Ischemia Detection System, Spectros Corp., Portola Valley, California, USA), | NA | 178 | NA | STO2 | NA | 6 | NA | 8 | | NA | not defined |  |
| Impellizzeri | 2020 | Italy | Retrospective Case Control | Single centre | 196 | 98 | 98 | 66 | 71 | 53 | 45 | 57 | 41 | NA | NA | Colorectal resections | (KARL STORZ GmbH & Co.KG, Tuttlingen, Germany) | 0 | NA | NA | ICG FA | 8 | 0 | 6 | NA | | NA | ISREC |  |
| Ishii | 2019 | Japan | Retrospective Case Control | Single centre | 488 | 223 | 265 | 67 | 69 | 126 | 97 | 136 | 129 | 22.9 | 22.7 | Colorectal resections | NA | 0 | NA | NA | ICG FA | 7 | 4 | 14 | NA | | NA | which was defined as discharge of feces or gas from the drain or wound, discharge of pus per rectum, or rectovaginal fistula |  |
| Iwamoto | 2020 | Japan | Prospective Cohort Study | Single centre | 25 | 25 | 0 | 68.16 | NA | 17 | 8 | NA | NA | 21.544 | NA | LAR | (Hamamatsu Photonics K.K., Hamamatsu, Japan). | NA | 259 | NA | ICG FA | 0 | 6 | NA | NA | | NA | ISREC |  |
| Jafari | 2013 | USA | Retrospective Case-control Analysis | Single centre | 38 | 16 | 22 | 58 | 63 | 12 | 4 | 16 | 6 | 27 | 27 | LAR | Olympus Corporation (Tokyo, Japan), Karl Storz GmbH (Tuttlingen, Germany), Stryker Corporation (Ontario, Canada).(Portage, MI, USA), and Novadaq Technologies | 0 | 285 | 264 | ICG FA | 3.04 | 6 | 18 | 1 | | 2 | not defined |  |
| Jafari | 2015 | USA | Prospective, Open-label, Clinical Trial | Multi-centre | 139 | 139 | 0 | 58 | NA | 74 | 65 | NA | NA | 29 | NA | AR and Left Colectomy | PINPOINT Endoscopic Fluorescence Imaging System (Novadaq). | NA | 240 | NA | ICG FA | 11 | 2 | NA | NA | | NA | Radiographic evidence |  |
| Jafari | 2021 | USA | Randomized, Controlled, Parallel Trial | Multi-centre | 347 | 178 | 169 | 57.2 | 57 | 109 | 69 | 99 | 70 | 27.8 | 28.2 | LAR | via PINPOINT and/or SPY Elite near infrared range fluorescence imaging (Stryker, Kalamazoo, MI) | NA | NA | NA | ICG FA | NA | 16 | 16 | NA | | NA | NA |  |
| Jansen-Winkeln | 2019 | Germany | Open Label, Single Armed, Non Randomised Trial | Single centre | 20 | 20 | 0 | 63 | NA | 11 | 9 | NA | NA | 25 | NA | minimally invasive colorectal resections for left/right hemiscolectomies, sigmoid + rectal resections | e TIVITA® Tissue System (Diaspective Vision GmbH, Am Salzhaff, Germany) | 0 | 290 | NA | HSI | 20 | 0 | NA | NA | | NA | Anastomotic leakage was defined as any type of disruption of the anastomosis, which was proven by direct signs, such as radiologic examination (CT scan with rectal enema), endoscopy (rigid or flexible), or rectal-digital examination. |  |
| Kaneko | 2020 | Japan | Case-Control Study | Single centre | 123 | 36 | 87 | 66 | 69 | 20 | 16 | 48 | 39 | NA | NA | sigmoidectomy, high anterior resection, low anterior resection, or ultra-low anterior resection | Softcare Co.,Ltd., Fukutsu, Fukuoka, Japan LSFG-PFI device | 0 | 368 | 312 | LSCI | 0 | 3 | 13 | 0 | | 2 | Therefore, we included only symptomatic AL for the examination in this study. Clinical signs of AL were defined as abdominal pain, fever, purulent or fecal discharge from the pelvic drain, peritonitis, and pelvic abscess. |  |
| Karliczek | 2010 | Holland | Observational Study | Single centre | 77 | 77 | 0 | 68 | NA | 42 | 35 | NA | NA | 26.81818 | NA | elective colorectal resection | VLS oximeter (T-Stat, model 303 Ischemia Detection System; Spectros Corp., Portola Valley, Califoria, USA) | 1 | 183.6364 | NA | STO2 | NA | 14 | NA | 11 | | NA | not defined |  |
| Kawada | 2017 | Japan | Prospective Cohort Study | Single centre | 68 | 68 | 0 | 66 | NA | 48 | 20 | NA | NA | 22.7 | NA | sigmoidectomy, high anterior resection, low anterior resection, or hartmann's | NIR camera system (PDE-neo system; Hamamatsu Photonics K.K., Hamamatsu, Japan) | NA | NA | NA | ICG FA | 18 | 3 | NA | 3 | | NA | AL was defined as any disruption of the anastomosis that was confirmed by digital rectal examination, sigmoidoscopy and radiographic examination + ISREC |  |
| Kim | 2016 | Korea | Prospective Case-Control | Single centre | 436 | 123 | 313 | 57 | 58 | 73 | 50 | 192 | 121 | 23.9 | 23.7 | LAR, UlAR, ISR | The da Vinci Si® high-definition (HD) vision system (Firefly ™, Intuitive Surgical, Sunnyvale, CA, USA) | NA | 164 | 186 | ICG FA | N/A | 1 | 17 | NA | | NA | AL was defined as any disruption of the anastomosis, including leakage, abscess and enteric fistula, verified by water-soluble contrast enema, pelvic computed tomography and clinical findings, and observed for at least 6 months after the take-down of diversion or anastomosis without diversion. |  |
| Kin | 2015 | USA | Case Matched Retrospective Study | Single centre | 346 | 173 | 173 | 58.2 | 58.1 | 54 | 46 | 54 | 46 | 27 | 26.5 | L colectomy, sigmoid colectomy, proctosigmoidectomy, low proctosigmoidectomy, ULproctosigmoidectomy | SPY Imaging System (Novadaq Technologies Inc, Bonita Springs, FL) | NA | NA | NA | ICG FA | NA | 13 | 11 | 54 | | 36 | 1) an anastomotic defect noted on physical examination, 2) an anastomotic defect confirmed in the operating room, 3) an anastomotic defect seen on proctoscopy, 4) radiologic evidence of a leak consisting of either a defect in the anastomosis and an adjacent fluid collection, or stranding or the extravasation of rectal contrast into the extraluminal space, or 5) clinical evidence of a leak such as feculent output from a pelvic drain. The secondary outcome was whether the results of intraoperative fluorescence angiography changed surgical management. |  |
| Kojima | 2019 | Japan | Pilot Study | Single centre | 8 | 8 | 0 | 68.25 | NA | 4 | 4 | NA | NA | 20.15 | NA | left colectomy, high ant resection, hartmann's, sigmoid colectomy | LSCI instrument (moorFLPI-2, Moor Instruments, Axminster, UK) | 0 | NA | NA | LSCI | NA | 0 | NA | 0 | | NA | Anastomotic leakage was defined as any disruption of the anastomosis occurring within 30 days of surgery as confirmed by a water-soluble contrast enema study. We focused only on symptomatic anastomotic leakage in the current analysis; we did not consider asymptomatic leakage because in our institution, contrast enema studies are not routinely performed after colorectal surgery in asymptomatic patients. |  |
| Kojima | 2020 | Japan | Case Control | Single centre | 54 | 27 | 27 | 70 | 72 | 15 | 12 | 14 | 13 | 20.1 | 21.3 | Left colectomy, sigmoid colectomy, ant resection, intersphincteric resection | (moorFLPI-2; Moor Instruments, Axminster, UK) | 0 | NA | NA | LSCI | NA | 0 | 5 | NA | | NA | Anastomotic leakage was defined as any disruption of the anastomosis occurring within 30 days of surgery as confirmed by a water-soluble contrast enema study. We focused only on symptomatic anastomotic leakage in the current analysis; we did not consider asymptomatic leakage because in our institution, contrast enema studies are not routinely performed after colorectal surgery in asymptomatic patients. |  |
| Kudszus | 2010 | Germany | Retrospective Case-Control Study | Single centre | 402 | 201 | 201 | 67.8 | 69 | 85 | 116 | 85 | 116 | 25.3 | 25.7 | right hemicolectomy, segmental resection, left hemicolectomy | (IC-View®, Pulsion Medical Systems AG, Munich, Germany). | NA | NA | NA | ICG FA | 28 | 7 | 15 | 7 | | 15 | not defined |  |
| Losurdo | 2020 | Italy | Retrospective, Propensity Score-Matched Cohort Study | Single centre | 272 | 177 | 95 | 69.9 | 67.9 | 109 | 68 | 37 | 58 | 25.6 | 27.3 | left colectomy and rectal resecations | SPIES system for a laparoscopic procedure (Karl Storz, Germany) | NA | 219.1 | 219.2 | ICG FA | 20 | 19 | 17 | 11 | | 15 | ISREC |  |
| Mizrahi | 2018 | USA | Retrospective Cohort Study | Single centre | 60 | 30 | 30 | 58 | 58 | 16 | 14 | 18 | 12 | 25.9 | 27.2 | LAR | PINPOINT | 0 | 347 | 347 | ICG FA | 4 | 0 | 2 | 0 | | 0 | ISREC |  |
| Ohya | 2020 | Japan | Retrospective Observational Study | Multi-centre | 400 | 400 | 0 | 74 | NA | 203 | 197 | NA | NA | 22.4 | NA | ileocaecal resection, right hemicolectomy, transverse colectomy, left hemicolectomy, sigmoidectomy | Karl Storz (D-light P; Tuttlingen, Germany) and Striker Corporation (1588 AIM Platform; Michigan, USA) | NA | 182 | NA | ICG FA | 11 | 10 | NA | 7 | | NA | abnormalities in the drain placed behind the anastomosis site when inserting a drain, and (2) fluid collection and/or an elevated CT value around the anastomotic site on abdominal CT performed for clinical symptoms, such as a fever and abdominal pain and increased inflammatory response. |  |
| Otero-Pineiro | 2021 | Spain | Comparative Study Based on a Retrospective Analysis of Prospectively Collected Data, | Single centre | 284 | 84 | 200 | 68 | 66.6 | 51 | 29 | 123 | 81 | 26.1 | 25.4 | HAR, LAR, ULAR, splenic flexure mobilisation | PINPOINT | NA | NA | NA | ICG FA | 23 | 2 | 23 | 6 | | 22 | Clinical AL was defined as a confirmed defect of the intestinal wall at the level of the anastomosis, which leads to intra and extraluminal communication, as proven by the following: anastomotic defect noted on digital rectal examination, or radiologic evidence of extravasation of rectal contrast that has an impact on patient management |  |
| Ris | 2014 | UK | Interventional Studies Without Concurrent Controls | Single centre | 30 | 30 | 0 | 64 | NA | 19 | 11 | NA | NA | 26.7 | NA | HAR, LAR, right hemicolectomy | PINPOINT | 0 | 176.6 | NA | ICG FA | 0 | 0 | NA | 0 | | NA | not defined |  |
| Ris | 2018 | Multicountry | Interventional Studies Without Concurrent Controls | Multi-centre | 504 | 504 | 0 | 64 | NA | 279 | 225 | NA | NA | 25 | NA | Right hemi, HAR, LAR, Hartmann's, ileoanal J pouch, Ileorectal anastomosis | PINPOINT | 0 | NA | NA | ICG FA | 29 | 12 | NA | 12 | | NA | not defined |  |
| Ryu | 2020 | Japan | Interventional Studies Without Concurrent Controls | Single centre | 50 | 50 | 0 | 70.5 | NA | 27 | 23 | NA | NA | 22.03 | NA | laparoscopic colorectal cancer surgery | (VISERA ELITE2 system; Olympus, Tokyo, Japan) | NA | NA | NA | ICG FA | 3 | 1 | NA | NA | | NA | not defined |  |
| Santi | 2018 | Italy | Interventional Studies Without Concurrent Controls | Single centre | 38 | 38 | 0 | 61 | NA | 18 | 20 | NA | NA | 26 | NA | Left colectomy, right colectomy, transverse resections, splenic flexure resections | IMAGE1 SPIES System (Karl Stortz GmbH & co KG, Tuttlingen, Germany) | NA | NA | NA | ICG FA | 1 | 1 | NA | NA | | NA | not defined |  |
| Shapera | 2019 | USA | Retrospective Case-Control | Single centre | 103 | 74 | 29 | 58 | 60 | 42 | 32 | 16 | 13 | 28 | 27 | LAR, Left hemicolectomy, sigmoidectomy | Da Vinci Xi robot (Intuitive Surgical), | 0 | NA | NA | ICG FA | 4 | 0 | 1 | 0 | | 1 | not defined |  |
| Skrovina | 2020 | Czech Republic | Prospective Non-Randomized Comparative Study | Single centre | 100 | 50 | 50 | 62.4 | 65 | 34 | 16 | 29 | 21 | 27 | 27 | low rectal resection for cancer with total mesorectal excision, | SPIES system (KARL STORZ GmbH & Co. KG, Tuttlingen, Germany) or the Firefly robotic surgical system da Vinci Xi (Intuitive Surgical, Sunnyvale, CA, USA) | 0 | 242 | 219 | ICG FA | 6 | 5 | 9 | NA | | NA | ISREC |  |
| Somashekhar | 2020 | India | Interventional Studies Without Concurrent Controls | Single centre | 50 | 50 | 0 | 54.52 | NA | 32 | 18 | NA | NA | 30 | NA | Ant Resection, LAR, ULAR | Da Vinci | 0 | NA | NA | ICG FA | 44 | 1 | NA | 0 | | NA | not defined |  |
| Spinelli | 2019 | Italy | Case Control | Multi-centre | 64 | 32 | 32 | 39.41 | 45.75 | 21 | 11 | 17 | 15 | 22.16 | 22.84 | Ileo-anal pouch formation | PINPOINT + SPIES + IMAGE1 | NA | 315.2 | 351.2 | ICG FA | NA | 0 | 1 | 1 | | 0 | not defined |  |
| Su | 2020 | China | Retrospective Case-Control | Single centre | 189 | 84 | 105 | 59.1 | 60.2 | 48 | 36 | 55 | 50 | 24.6 | 23.8 | right colectomy, transverse colectomy, left colectomy | opto-cam 2100 (Optomedic, Guangdong, China) | 0 | 125.8 | 136.6 | ICG FA | 4 | 0 | 0 | 0 | | 0 | not defined |  |
| Tokunaga | 2021 | Japan | Interventional Studies Without Concurrent Controls | Single centre | 45 | 45 | 0 | 64 | NA | 26 | 19 | NA | NA | NA | NA | HAR + LAR | HyperEye Medical System (HEMS; Mizuho Medical Co. Ltd, Tokyo, Japan). + thermal camera (Nippon Avionics, Tokyo, Japan) | 0 | 253 | NA | ICG FA | NA | 3 | NA | 1 | | NA | not defined |  |
| Tsang | 2020 | China | Case-Control Study | Single centre | 131 | 63 | 68 | 69.82 | 67.71 | 39 | 23 | 47 | 22 | 23.47 | 22.36 | Right hemi, left hemi, ant resection, LAR/TME | da Vinci Xi robotic surgical  system (Intuitive Surgical, Sunnyvale, CA, USA) or Olympus  laparoscopic camera system OTV-S300 | 0 | 178.95 | 140.84 | ICG FA | 1 | 2 | 3 | 2 | | 3 | Anastomotic leakage was defined  as disruption of bowel wall integrity at the anastomosis  with communication between intra- and extraluminal compartments.  Perianastomotic abscess was regarded as leakage  as well. |  |
| Wada | 2017 | Japan | Interventional Studies Without Concurrent Controls | Single centre | 112 | 112 | 0 | 67 | 0 | 81 | 31 | NA | NA | 22.8 | NA | HAR, LAR, sigmoid resection | (PDE-neo System; Hamamatsu Photonics  K.K., Hamamatsu, Japan) | 0 | NA | NA | ICG FA | 18 | 5 | NA | 5 | | NA | AL was defined as any disruption of the anastomosis confirmed by digital rectal examination, sigmoidoscopy, and radiographic examination  (e.g., extravasation of endoluminally administered watersoluble  contrast enema, abscess at the level of anastomosis,  and fluid/air bubbles surrounding the anastomosis), as  previously described |  |
| Watanabe | 2015 | Japan | Interventional Studies Without Concurrent Controls | Single centre | 119 | 119 | 0 | 68.4 | NA | 83 | 36 | NA | NA | 22.5 | NA | partial colectomy (descending colon), sigmoidectomy, HAR, LAR, ULAR, Hartmann) | Olympus Medical  Systems Corporation (Tokyo, Japan) and Mizuho Corporation  (Tokyo, Japan). | NA | NA | NA | ICG FA | NA | 7 | NA | NA | | NA | anastomotic leakage  was demonstrated radiologically using a contrast enema. |  |
| Watanabe | 2020 | Japan | Retrospective Case-Control Study | Multi-centre | 550 | 236 | 314 | 66 | 66 | 128 | 83 | 131 | 80 | 22.3 | 22.4 | Laparocopic LAR for rectal Cancer | Karl Storz/Stryker | NA | 219 | 214 | ICG FA | 12 | 16 | 42 | 2 | | 10 | not defined |  |
| Wojcik | 2020 | France | Prospective Case Matched Control Study | Single centre | 111 | 46 | 65 | 65.7 | 68.6 | 30 | 16 | 40 | 25 | 25.8 | 26.2 | left colectomy or anterior resection | (FLUOBEAM; Fluoptics, Grenoble, France) or on  fusion images merging NIR and standard white light  images (PINPOINT; Stryker, Kalamazoo, Michigan,  USA). | NA | 215 | 207 | ICG FA | NA | 3 | 11 | NA | | NA | ISREC |  |
| Yanagita | 2021 | Japan | Retrospective Review of Prospective Data With Matched Control Group | Single centre | 384 | 197 | 187 | 70 | 69 | 116 | 81 | 115 | 72 | 22.3 | 22 | Left hemi, sigmoidectomy, HAR, LAR, ULAR, | Hyper Eye Medical Systems: Mizuho Medical Co.,  Ltd, Nagoya, Japan and/or IMAGE 1 SPIES™, KARL  STORZ SE & Co. KG, Tuttlingen, Germany) | NA | 288 | 257 | ICG FA | 18 | 3 | 10 | NA | | NA | ISREC |  |
| Ogino | 2018 | Japan | Interventional Studies Without Concurrent Controls | Single centre | 74 | 74 | 0 | 71 | NA | 40 | 34 | NA | NA | 20.6 | NA | Right colectomy, Left colocetomy, AR, LAR, Hartmann's, abdominoperineal resection | | NA | 243 | NA | ICG FA | 6 | 1 | NA | 0 | | NA | Only symptomatic AL was included in the analysis. |  |
| Sherwinter | 2021 | USA | Interventional Studies Without Concurrent Controls | Multi-centre | 32 | 32 | 0 | 64 | NA | 18 | 14 | NA | NA | 42.3 | NA | Right hemi left hemi, sigmoidectomy, LAR | IntraOx device (ViOptix Inc, Newark, CA USA) Spy-phi system (Stryker, Kalamazoo, MI, USA) (Video 1). | NA | NA | NA | STO2 | 3 | 0 | NA | NA | | NA | not defined |  |
| Jansen-Winkeln | 2021 | Germany | Interventional Studies Without Concurrent Controls | Single centre | 30 | 30 | 0 | 59.5 | NA | 21 | 11 | NA | NA | 29 | NA | right hemicolectomy, sigmoid or rectal resections | TIVITA® Tissue system (Diaspective Vision GmbH, Am Salzhaff, Germany), FA VisionSense-3 iridium camera system (Medtronic GmbH, Meerbusch, Germany) | NA | 256 | NA | HSI | 10 | 1 | NA | NA | | NA | CD 3 |  |
| Janse-Winkeln | 2022 | Germany | Interventional Studies Without Concurrent Controls | Single centre | 115 | 115 | 0 | 63 | NA | 74 | 41 | NA | NA | 26 | NA | left hemicolectomy (including sigmoid resection) (n = 49), rectal resection (n = 54)—48 with protective ileostomy, and right hemicolectomy (n = 12). | TIVITA® Tissue system (Diaspective Vision GmbH, Am Salzhaff, Germany),# | NA | 225 | NA | HSI | NA | 8 | NA | 0 | |  | CD3 |  |
| Yoshinaka | 2022 | Japan | Interventional Studies Without Concurrent Controls | Single centre | 73 | 73 | 0 | 70 | NA | 51 | 22 | NA | NA | 22 | NA | left hemicolectomy,  sigmoidectomy, high anterior resection, low anterior resection  or very low anterior resection was selected. | Vivo Optical Spectroscopy (INVOS™) system (Medtronic, Minneapolis, MN, USA). | NA | 255-315 | NA | STO2 | NA | 6 | NA | NA | | NA | Not included |  |
| Polom | 2022 | Poland | Interventional Studies Without Concurrent Controls | Multi-centre | 12 | 12 | 0 | 68.3 | NA | 9 | 3 | NA | NA | 27 | NA | Right hemicolectomy 2 (16.7%) Rectum anterior resection 5 (41.7%) Rectum anterior resection + ileostomy 2 (16.7%) Hartmann operation 1 (8.3%) Abdominal sacral resection | The Quest Artemis (Quest Medical Imaging, Middenmeer, The Netherlands) | 0 | NA | NA | ICG FA | 0 | 0 | NA | NA | | NA | CD |  |
| Pfahl | 2022 | Germany | Interventional Studies Without Concurrent Controls | Single centre | 46 | 46 | 0 | 63 | NA | NA | NA | NA | NA | NA | NA | Colorectal resections | TIVITA  Suite (Diaspective Vision GmbH, Am Salzhaff, Germany) and ImageJ (Wayne Rasband and  contributors, National Institutes of Health, USA). | 0 | NA | NA | HSI | NA | 6 | NA | NA | | NA | CD |  |
| Neddermeyer | 2022 | Germany | Retrospective Case Control Study | Single centre | 132 | 70 | 62 | 66.5 | 59.5 | 48 | 22 | 39 | 23 | 26.7 | 26.7 | 70 sigmoid resections  and 62 total mesorectal excisions (TMEs)], | PINPOINT endoscope Fluorescence Imaging System (Novadaq,  Ontario, Canada), | 0 | 222.5 | 214 | ICG FA | 9 | 10 | 9 | 0 | | 3 | CD |  |
| Gomez Rosardo | 2022 | Spain | Interventional Studies Without Concurrent Controls | Single centre | 70 | 70 | 0 | 65.8 | NA | 44 | 26 | NA | NA | 27.4 | NA | L + R resections with anastomoses | VisionSense™ VS Iridium (Medtronic, Mansfield, MA, USA) | 0 | 165.7 | NA | ICG FA | 16 | 9 | NA | NA | | NA | CD |  |
| Barberio | 2022 | Italy | Interventional Studies Without Concurrent Controls | Single centre | 54 | 54 | 0 | 66 | NA | 36 | 16 | NA | NA | NA | NA | Fifteen patients  underwent oncological left hemicolectomies, 9 patients had  tubular sigmoid colon resections, 25 patients had anterior  rectal resection, one patient underwent a transanal total mesorectal  excision (TaTME), and 2 patients had a Hartmann  procedure reversal. | (TIVITA®, Diaspective Vision, Am Salzhaff, Germany) | 0 | 178 | NA | HSI | NA | 6 | NA | NA | | NA | CD |  |
